# Survival Strategies of *Streptococcus pyogenes* in Response to Phage Infection

**DOI:** 10.3390/v13040612

**Published:** 2021-04-02

**Authors:** Dior Beerens, Sandra Franch-Arroyo, Timothy J. Sullivan, Christian Goosmann, Volker Brinkmann, Emmanuelle Charpentier

**Affiliations:** 1 Max Planck Unit for the Science of Pathogens, 10117 Berlin, Germany; beerens@mpusp.mpg.de (D.B.); arroyo@mpusp.mpg.de (S.F.-A.); Timothy.J.Sullivan@darthmouth.edu (T.J.S.); 2 Institute for Biology, Faculty of Life Sciences, Humboldt University Berlin, 10115 Berlin, Germany; 3 Core Facility Microscopy, Max Planck Institute for Infection Biology, 10117 Berlin, Germany; goosmann@mpiib-berlin.mpg.de (C.G.); brinkmann@mpiib-berlin.mpg.de (V.B.)

**Keywords:** *Streptococcus pyogenes*, bacteriophage defense, Phage A1, CRISPR-Cas, membrane vesicles, capsule

## Abstract

Bacteriophages exert strong evolutionary pressure on their microbial hosts. In their lytic lifecycle, complete bacterial subpopulations are utilized as hosts for bacteriophage replication. However, during their lysogenic lifecycle, bacteriophages can integrate into the host chromosome and alter the host’s genomic make-up, possibly resulting in evolutionary important adjustments. Not surprisingly, bacteria have evolved sophisticated immune systems to protect against phage infection. *Streptococcus pyogenes* isolates are frequently lysogenic and their prophages have been shown to be major contributors to the virulence of this pathogen. Most *S. pyogenes* phage research has focused on genomic prophages in relation to virulence, but little is known about the defensive arsenal of *S. pyogenes* against lytic phage infection. Here, we characterized Phage A1, an *S. pyogenes* bacteriophage, and investigated several mechanisms that *S. pyogenes* utilizes to protect itself against phage predation. We show that Phage A1 belongs to the *Siphoviridae* family and contains a circular double-stranded DNA genome that follows a modular organization described for other streptococcal phages. After infection, the Phage A1 genome can be detected in isolated *S. pyogenes* survivor strains, which enables the survival of the bacterial host and Phage A1 resistance. Furthermore, we demonstrate that the type II-A CRISPR-Cas system of *S. pyogenes* acquires new spacers upon phage infection, which are increasingly detectable in the absence of a capsule. Lastly, we show that *S. pyogenes* produces membrane vesicles that bind to phages, thereby limiting the pool of phages available for infection. Altogether, this work provides novel insight into survival strategies employed by *S. pyogenes* to combat phage predation.

## 1. Introduction

The so-called “flesh-eating bacterium” *Streptococcus pyogenes* is a Gram-positive β-hemolytic pathogen that strictly infects humans in natural settings [1,2]. Although *S. pyogenes* can colonize the throat and skin asymptomatically [3], the hallmark of *S. pyogenes* is its ability to cause a vast variety of diseases [4]. Clinical manifestations of *S. pyogenes*-infections range from superficial skin infections and pharyngitis to toxin-mediated diseases (e.g., streptococcal toxic shock syndrome (STSS)), and invasive disease in subcutaneous tissues [5].

With an estimated 616 million pharyngitis cases, almost 2 million severe *S. pyogenes* disease cases and more than 500 000 deaths per year, *S. pyogenes* remains among the top 10 individual pathogens causing morbidity and mortality [6]. To date, *S. pyogenes* has remained universally susceptible to penicillin, which remains the drug of choice to treat pharyngeal infections as well as for complicated or invasive infections [7]. Although no penicillin-resistant *S. pyogenes* strains have been observed in clinical settings, up to 40% treatment failure of severe *S. pyogenes*-associated disease with these antibiotics has been reported [8]. These high mortality and treatment failure rates—despite the use of adequate antimicrobials—call for effective alternatives. One alternative to antibiotics that has recently gained an increasing momentum is phage therapy [9]. However, bacteriophages are known to play important roles in the shaping of bacterial populations as well as the dissemination of bacterial genetic material to new strains, potentially resulting in the spread of virulence factors and antibiotic resistance genes [10].

The M protein is considered a major virulence determinant for *S. pyogenes* [11], since *emm* gene knock-out (KO) mutants are neither able to survive in mouse models nor in human phagocyte-containing blood [12,13]. Additionally, M protein hyperproduction inhibits phage adsorption, which results in phage-resistant *S. pyogenes* mutants [14], suggesting that the M protein could serve a dual function in both phage and phagocyte resistance. Another important virulence factor of *S. pyogenes* is the hyaluronic acid (HA) capsule, which is chemically identical to HA produced by mammalian tissues [15]. This molecular mimicry between the bacterial surface and host molecules reduces the ability of the host immune system to detect and kill *S. pyogenes*. Indeed, encapsulated *S. pyogenes* strains are associated with more severe disease [16] and were shown to be more resistant to neutrophil-mediated killing in mice [17]. Early studies have also shown that enzymatic removal of the HA capsule increased phage infectivity, suggesting that it provides a barrier between bacteriophages and their bacterial surface-based receptor [18]. The biosynthesis of the HA capsule is regulated by the *hasABC* operon [19], but only HasA and HasB are required for the production of the HA capsule [20]. The *hasABC* operon is regulated by the Control of Virulence (CovRS) two-component system [21], and spontaneous mutations within *covRS* result in highly encapsulated and invasive *S. pyogenes* strains [22].

*S. pyogenes* isolates are frequently lysogenic, and most *S. pyogenes* phage research has focused on genomic prophages in relation to horizontal gene transfer (HGT) and virulence, but surprisingly little is known about the interplay between *S. pyogenes* and its lytic phages [23]. The few reported lytic phages include Phage A1, A6, A12, A25 and A27; however, only Phage A25 has been studied in detail to date [18,24,25]. Phage A25 belongs to the *Siphoviridae* family [26], with low burst sizes ranging from 12 to 32 plaque-forming units (PFUs) per infected cell depending on the host strain [27,28]. Host recognition is thought to be a two-step process, as peptidoglycan was shown to be the cell receptor for Phage A25 that mediated reversible adsorption, but an unknown additional factor is required for irreversible binding and DNA injection [29]. Lytic *S. pyogenes* Phages A6, A12, A25 and A27 have a broad host range and are able to infect some strains of Group C Streptococci (GCS), and Phage A25 was shown to be adaptable to Group G Streptococci (GGS) [25,29]. Recently, the genome sequence of Phage A25 has been determined to be 33,900 bp in size with a GC content of 38.44% [30]. The phages of *S. pyogenes* comprise a modular genetic arrangement with regions dedicated to gene regulation, DNA replication, DNA cleavage, DNA packaging, capsid and tail morphogenesis and lysis. In contrast to most prophages of *S. pyogenes*, however, no identifiable virulence genes were found in the genome of Phage A25 [30]. Genomic analysis of the Phage A25 and prophage genomes provided hints towards the broad host range of *S. pyogenes* phages. A highly mosaic nature of these genomes was identified, and genetic modules were shown to be shared between phages of *S. pyogenes*, *Streptococcus pneumoniae* and *Streptococcus suis*, among others [30].

In this study, we demonstrate that Phage A1 is not a strictly lytic phage as previously reported and we shed light on immune strategies of *S. pyogenes* to withstand phage infection. We observe that membrane vesicles (MVs) produced by *S. pyogenes* are able to scavenge phages from the environment and reduce the infection pressure. Additionally, *S. pyogenes* produces a hyaluronic acid capsule that prevents phages to bind to their receptor. Interestingly, CRISPR-Cas-mediated spacer acquisition is increased in the absence of the capsule. In a minor fraction of infected cells, the Phage A1 genome is detectable in the *S. pyogenes* genome, which enables the survival of the bacterial host. Phage A1-surviving clones neither become more virulent than their WT counterpart in vitro nor develop ampicillin tolerance. Together, our results highlight the complex interplay between streptococcal phages and their hosts, and provide insight into a potential role for streptococcal phages or phage-derived products for the treatment of severe infections.

## 2. Materials and Methods

### 2.1. Bacterial Strains

The bacterial strains used in this study are listed in Table 1. *S. pyogenes* strains were grown in either Todd Hewitt Broth (THB) or Brain Heart Infusion broth (BHI) without shaking at 37 °C and 5% CO_2_. TSA blood agar (TrypticaseTM Soy Agar, Becton Dickinson, Heidelberg, Germany) supplemented with 3% defibrinated sheep blood (Xebios Diagnostics, Düsseldorf, Germany) was used for cultivation on solid medium.

### 2.2. Phage Propagation

*S. pyogenes* ATCC 12202 was grown overnight and diluted 1:100 into 25 mL of fresh pre-warmed THB and incubated at 37 °C until mid-logarithmic phase (OD_600 nm_ = 0.25) was reached. At this point, CaCl_2_ was added to the culture to reach a final concentration of 10 mM. Phage A1 (ATCC 12202-B1) was added to achieve a final multiplicity of infection (MOI) of 0.1. After 20 min of incubation at 30 °C, the complete solution was transferred into 100 mL fresh 10 mM CaCl_2_-containing THB and incubated for an additional 4 h at 37 °C. Solid NaCl was added to reach a final concentration of 1 M. The solution was stored overnight at 4 °C and subsequently spun for 10 min at 10 000× *g* at 4 °C to pellet bacterial debris. The supernatant was filtered using a 0.22 μm filter and subjected to ultracentrifugation for 2 h at 50 000× *g* at 4 °C (SW32 Ti rotor, Optima XPN-100 ultracentrifuge, Beckmann Coulter, Krefeld, Germany). The resulting pellet was resuspended in SM-buffer (100 mM NaCl, 8 mM MgSO_4_, 50 mM Tris-HCl (pH 7.5)) and stored at 4 °C. Phage concentrations were determined by plaque assays on double-layer BHI plates.

### 2.3. Bacteriophage A1‒Membrane Vesicle Interaction Assays

*S. pyogenes* MVs were purified from late-logarithmic phase cultures (OD_600 nm_ = 0.4) according to a previously described method [35]. Briefly, bacterial cultures were centrifuged first (9300× *g*, 10 min, 10 °C), after which the supernatants were transferred to new tubes and centrifuged again to remove residual bacterial debris (18 600× *g*, 15 min, 10 °C). Supernatants were filtered twice through 0.22 µm pore polyethylsulfone (PES) membranes (Thermofisher scientific, Darmstadt, Germany) and spotted on TSA blood plates to confirm their sterility. These batches were centrifuged at 175,000× *g* for 4 h at 10 °C (Ti45 rotor, Optima XPN-100 ultracentrifuge, Beckmann Coulter). The resulting pellets were resuspended in 30 mL total volume phosphate-buffered saline (PBS) (Thermofisher scientific, Darmstadt, Germany), pooled, and centrifuged again at 175,000× *g* for 3 h at 10 °C. The resulting pellet was resuspended in a final volume of 1 mL of PBS prior to quantification using Nanoparticle Tracking Analysis (NTA) (NS300, Malvern Panalytical, Kassel, Germany).

MVs were mixed with 10 µL of Phage A1 in MOI ratios of 10, 1, 0.1:1000, respectively, and added to 80 µL 10 mM CaCl_2_-containing THB. After 15 min of incubation, samples were analyzed by electron microscopy (EM) as described below or added to a mid-log culture of *S. pyogenes* strains SF370 (OD_600 nm_ = 0.25) achieving a final MOI of 1, of which 200 µL was transferred into 96-well plates. The OD_600 nm_ was measured at 37 °C and 5% CO_2_ every 10 min for 4 h in a Synergy H1 microplate reader (BioTek, Bad Friedrichshall, Germany). A control consisted of MVs alone, where SM-buffer substituted phage samples were compared to pure phage infection. All experiments were performed in both technical and biological triplicates.

### 2.4. Electron Microscopy and Image Analysis

Overnight cultures of *S. pyogenes* ATCC 12202 or SF370 were diluted 1:100 in fresh THB medium and incubated until mid-logarithmic phase (OD_600 nm_ = 0.25) was reached. CaCl_2_ was supplied to the culture to reach a final concentration of 10 mM. Phage A1 was added to achieve an MOI of 10 and fixed with 2.5% glutaraldehyde (GA) 5, 15, 25, 45, 60 and 90 min post-infection. For scanning electron microscopy (SEM), bacteria were added directly onto poly-L-lysine-treated glass coverslips. The samples were post-fixed in 0.5% osmium-tetroxide, tannic acid and osmium-tetroxide again. The coverslips were then dehydrated in a graded ethanol series, dried in carbon dioxide at critical point and vacuum coated with 3 nm carbon-platinum. Imaging was performed using a LEO 1550 (Zeiss, Oberkochen, Germany) scanning electron microscope using an in-lens detector at 20 kV acceleration voltage. For transmission electron microscopy (TEM), aliquots of phage or phage:MV samples were applied to fresh glow discharged carbon-film-coated copper grids and allowed to adsorb for 10 min. After three washes with distilled water, the grids were contrasted with 4% phospho-tungstic-acid/1% trehalose, touched on filter paper and air-dried. The grids were examined in a LEO 906 (Zeiss AG, Oberkochen, Germany) electron microscope operated at 100 kV and images were recorded with a Morada digital camera (SIS-Olympus, Münster, Germany). Images were processed and analyzed with Fiji, an open source scientific image processing application (http://fiji.sc (accessed on 2 April 2021)).

### 2.5. One-Step Growth Analysis

One-Step Growth Curves (OSGCs) were performed to determine the latency period and burst size of Phage A1 according to a previously described method [36]. Briefly, a mid-log (OD_600 nm_ = 0.25) culture of *S. pyogenes* ATCC 12202 in 10 mM CaCl_2_-containing THB was infected with Phage A1 at an MOI of 1 and was allowed to adsorb for 10 min at 37 °C. The infected culture was centrifuged (4000× *g* for 3 min at RT, Heraeus Multifuge X3R, Thermo Scientific) to remove not adsorbed phages and the pellet was resuspended in 10 mL of pre-warmed 10 mM CaCl_2_-containing THB. A total of 100 µL of this culture was transferred into 9.9 mL of fresh 10 mM CaCl_2_-containing THB and gently mixed. A 10-fold serial dilution of this mixture was prepared twice and incubated at 37 °C and 5% CO_2_. Then, 100 µL samples were collected every 5–10 min (depending on the growth phase identified by initial trials) from each flask for PFU determination by plaque assays on double-layer BHI plates. *S. pyogenes* ATCC 12202 grown for 24 h in BHI served as plating host. Plates were incubated at 37 °C and 5% CO_2_ overnight. Burst size was calculated by dividing the average PFU after phage burst by the average amount of infection centers in the latent phase. The latent period was determined by the moment the first increase in PFU/mL was observed. OSGCs were performed in biological triplicates.

### 2.6. Spectrophotometric Phage A1 Lysis Profile

Overnight cultures of *S. pyogenes* were diluted 1:100 in fresh THB medium and incubated until mid-logarithmic phase (OD_600 nm_ = 0.25) was reached. CaCl_2_ was added to the culture to reach a final concentration of 10 mM. In total, 180 µL per condition was aliquoted in wells of a 96-well plate, and Phage A1 was added to achieve MOIs ranging from 1 to 0.001, after which the OD_600 nm_ of each culture was measured at 37 °C and 5% CO_2_ every 10 min for 4 h in a Synergy H1 microplate reader (BioTek, Bad Friedrichshall, Germany). Averages and standard deviations are given for one out of three biological replicates in triplicate per strain.

### 2.7. Phage A1 Efficiency of Plating on M1 Serotype S. pyogenes

Efficiency of plaquing (EOP) assays were performed by spotting serial dilutions of Phage A1 onto M1 serotype *S. pyogenes*. Tested *S. pyogenes* strains were diluted 1:100 in fresh THB medium and incubated until mid-logarithmic phase (OD_600 nm_ = 0.25) was reached. Hyaluronidase was added in a final concentration of 68 µg/mL when necessary. CaCl_2_ was added to the culture to reach a final concentration of 10 mM. A total of 250 µL per culture was mixed with 4 mL of 10 mM CaCl_2_-containing 0.7% BHI soft agar (48 °C) and directly poured onto pre-warmed BHI plates. After 15 min of solidification, 10 μL of a 10-fold dilution series of Phage A1 was spotted onto the soft agar and allowed to soak into the agar for 15 min. Plates were incubated overnight at 37 °C and 5% CO_2_. Counted plaques were converted to PFU/mL.

### 2.8. Lysogeny and CRISPR Acquisition Testing

*S. pyogenes* strains in mid-log phase were infected with Phage A1 at an MOI of 0.1 and were incubated at 37 °C and 5% CO_2_ for 24 h. The pellet of 1 mL lysed culture was resuspended in 100 µL fresh THB and plated on BA plates. After overnight growth, single colonies were selected, passaged twice in THB and tested for Phage A1 resistance. Phage resistance was examined by adding Phage A1 reaching MOIs of 0.1, 1, 10 and 100 to a mid-log culture of lysogenized *S. pyogenes* strains in the presence of Ca^2+^. Infected cultures were incubated for 4 h at 37 °C and 5% CO_2_ and pictured. The genomic DNA of resistant colonies was subjected to PCR to screen for phage integration with primers specific to Phage A1 (5′ GGGGGATAAAAATGAATGAAACGCT (**F**) and 5′ TGCGGACACTGACAAAATTTTTGG (**R1**)) and primers that should not amplify an amplicon when the Phage DNA is integrated (5′ GGGGGATAAAAATGAATGAAACGCT (**F**) and 5′ CGAATTTAGGAAGTTAATTTAGG (**R2**)). Primers that amplify the leader proximal part of the CRISPR array until the first spacer already present in the native CRISPR array were used to investigate CRISPR acquisition (Fw: 5′ GCTTTTCAAGACTGAAGTCTAGC and Rv: 5′ GCGCAAGAAGAAATCAACCAG).

### 2.9. Genomic DNA Isolation

Phage A1 lysates were subjected to DNAse I (2 U/mL final concentration) and RNaseA (100 μg/mL final concentration) treatment for 30 min at 37 °C. EDTA was added to a final concentration of 5 mM, mixed and incubated for 10 min at 70 °C. Hereafter, genomic DNA from 10^9^ PFUs was extracted using a Phage DNA Isolation Kit according to the manufacturer’s protocol (Norgen Biotek corp., Thorold, Canada). Genomic DNA from *S. pyogenes* was isolated using the NucleoSpin^®^ Microbial DNA kit (Macherey-Nagel, Düren, Germany) and purified with a genomic DNA Clean & Concentrator kit (Zymo research, Freiburg, Germany). Both Phage A1 and *S. pyogenes* DNA concentrations were determined by NanoDrop and DNA integrity was verified in a 0.7% agarose gel.

### 2.10. Genome Sequencing and Bioinformatic Analysis

A total of 750 ng of Phage A1 genomic DNA was used to generate the DNA library that was sequenced with 150 bp paired-ends using a NovaSeq 6000 system (Illumina Inc., San Diego, CA, USA). Whole Genome Sequencing (WGS) reads were trimmed using Trimmomatic [37] version 0.38. Trimmomatic was supplied with the following parameters: {ILLUMINACLIP:$ADAPTORS:2:30:10 LEADING:3 TRAILING:3 SLIDINGWINDOW:4:15 MINLEN:25 HEADCROP:3}, where $ADAPTORS is a fasta file containing TruSeq adapters and the sequence of the PhiX sequencing control. Trimmed read fastq files were down sampled to 250,000 paired-end reads using the linux utility ‘head –n 1000000’. Contigs were assembled using Spades [38] version 3.12.0 in paired-end mode with default parameters. The resulting viral genome was annotated using Prokka [39] version 1.13.7. Prokka was supplied with the following parameters: {--kingdom Viruses--proteins KT388093.1.gb--prefix phage}. Remaining hypothetical proteins were manually investigated for homology using BlastP. Phage A1 genome accession number: MW495853.

### 2.11. Phylogenetic Analysis

Streptococcal phage genomes were downloaded from the NCBI database and phylogenetic studies based on whole phage genomes were performed using VICTOR [40]. All pairwise comparisons of the nucleotide sequences were conducted using the Genome-BLAST Distance Phylogeny (GBDP) method under settings recommended for prokaryotic viruses [40]. The branch lengths are scaled in terms of the GBDP distance formula D_0_ [40]. Branch support was inferred from 100 pseudo-bootstrap replicates each. Trees were rooted and visualized with FigTree v1.4.4.

### 2.12. Cell Line Maintanance and Differentiation

The B-cell Leukaemia C/EBPα (CCAAT-enhancer-binding protein α, where CCAAT stands for a Cytosine-Cytosine-Adenosine-Adenosine-Thymidine motif) Estrogen Receptor clone 1 cell line (BLaER1) [41] was kindly provided by Prof. Thomas Graf (Centre for Genomic Regulation, Barcelona). BLaER1 cells were maintained in RPMI medium supplemented with 1% L-Glutamine, 1% penicillin-streptomycin (P/S, Sigma-Aldrich, Darmstadt, Germany), 10% FBS and 1% sodium pyruvate (Thermofisher scientific, Darmstadt, Germany). BLaER1 cells were transdifferentiated into monocyte-like cells for 7 days in six-well plates by seeding 1 × 10^6^ cells/well in 3 mL of medium supplemented with 10 ng/mL of human recombinant IL-3, 10 ng/mL of human recombinant Macrophage Colony-stimulating Factor 1 (both from PeproTech, Hamburg, Germany) and 100 nM of β-Estradiol (Sigma-Aldrich, Darmstadt, Germany). Half of the medium was exchanged at days 2 and 5 for fresh medium containing identical supplements. On day 7 after differentiation, cells were harvested, counted with a CASY cell counter and cells were spun down (500× *g*, 5 min, RT). Cell pellets were resuspended in an adjusted volume of fresh medium without cytokines or P/S at a final density of 1 × 10^6^ cells/mL. Finally, 10^6^ cells were seeded per well in a 24-well plate for infection assays.

### 2.13. Infection Assay

*S. pyogenes* strains in late-log phase (OD_600 nm_ = 0.4) were spun down (6000× *g*, 3 min, RT) and washed once with 1 mL of phosphate-buffered saline (PBS). Bacterial suspensions were passed 10 times through a 27 Gauge syringe insert (NeoLab, Heidelberg, Germany) in a 1 mL syringe (VWR) to separate the cocci chains, and 10^6^ BLaER1 cells were infected at MOI 1. Plates were centrifuged (300× *g*, 5 min, RT) to synchronize the infection. P/S was added after 1 h to a final concentration of 1% to inhibit bacterial replication and excessive cell death. After overnight incubation, plates were centrifuged (500× *g*, 5 min, RT), after which supernatants were collected and stored at −20 °C until use. Supernatants were thawed on ice and IL-6 and IL-1β concentrations were measured using Enzyme-Linked ImmunoSorbent Assay (ELISA) kits (Thermofisher Scientific, Darmstadt, Germany) according to the manufacturer’s protocol. ELISA data were analyzed by extrapolating values of each sample from the standard curve using a log-log fit. The standard curve was generated for each plate by plotting the logarithm of the absorbance of the standards against the logarithm of the concentrations of each standard. In contrast, fresh supernatants were used to measure cell death using the Lactate Dehydrogenase (LDH)-Cytotoxicity Assay Kit II (Abcam, Cambridge, UK) according to the manufacturer’s instructions.

### 2.14. Antibiotic Susceptibility

The susceptibility of *S. pyogenes* and their lysogenized variants to the commonly used antibiotic ampicillin to treat *S. pyogenes* infections was tested. *S. pyogenes* strains were grown on BA plates and harvested in sterile PBS. *S. pyogenes* suspensions were set at ~0.5 McFarland and swabbed onto Mueller-Hinton II agar (MH-II) plates (Sigma-Aldrich, Darmstadt, Germany). Ampicillin E-tests (bestbion dx, Köln, Germany) were placed in the middle of the plate and plates were incubated overnight at 37 °C with 5% CO_2_. The minimum inhibitory concentration (MIC) was determined after 24 h.

### 2.15. Statistics

Paired *t* tests or one-way ANOVA tests were performed for comparisons between data sets. All analyses were performed using Prism GraphPad software v8.0.0 (GraphPad software Inc, San Diego, CA, USA). *p* < 0.05 was considered statistically significant.

## 3. Results

### 3.1. Virion Morphology and Physiological Characterization of S. pyogenes Bacteriophage A1

**Figure 1:**
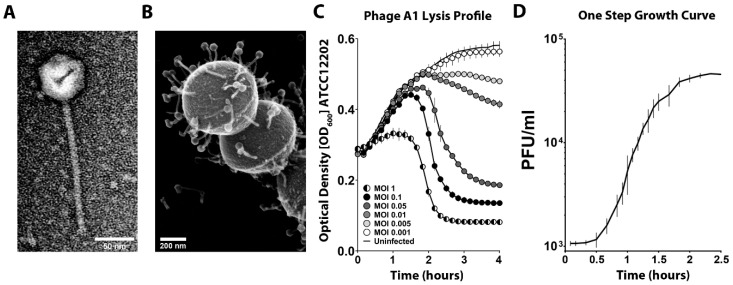
Phage A1 morphology and growth characteristics. (**A**) TEM micrograph of purified Phage A1; (**B**) SEM micrograph of Phage A1 bound to *S. pyogenes* ATCC 12202 at multiplicity of infection (MOI) 25 after 10 min of coincubation; (**C**) Bacteriolytic activity of Phage A1 on *S. pyogenes* ATCC 12202 infected at different MOIs over time. Each curve shows mean values ± SD of one out of three experiments in triplicates; (**D**) One-step growth curves (OSGCs) of Phage A1 on *S. pyogenes* ATCC 12202. Mean values ± SD from three independent experiments are shown.

*S. pyogenes* Bacteriophage A1 (Phage A1) was obtained from the ATCC database and, after obtaining high titer phage stocks, the morphology of Phage A1 was investigated by both TEM and SEM. The obtained EM images revealed that Phage A1 virions have icosahedral heads and long non-contractile tails (Figure 1A,B), typical for the *Siphoviridae* family among the *Caudovirales* order. In total, 30 isolated phages in TEM images were measured to determine phage dimensions, which are summarized in Table 2. We continued with the investigation of the physiological properties of Phage A1. First, we examined the lysis profile of Phage A1 on *S. pyogenes* strain ATCC 12202 (Figure 1C). Phage A1 exhibited a strong lytic phenotype at MOI 1 and this lytic phenotype was still present at MOI 0.05 with rapid lysis of the culture occurring after 1.5 h of infection. However, we observed a clear delay in lysis or no lysis occurring at MOI 0.01 or lower. Additionally, cultures were not cleared after overnight incubation at lower MOIs starting from MOI 0.01. We performed one-step growth curves (OSGCs) to determine the lag phase and average number of newly produced phages per infected cell. Phage A1 showed a latent period of 40 min, after which the first increase in PFUs was observed. Additionally, the burst size of Phage A1 is approximately 31 PFUs per infected cell on strain ATCC 12202 (Figure 1D).

### 3.2. Genome Sequence and Phylogeny of S. pyogenes Bacteriophage A1

**Figure 2:**
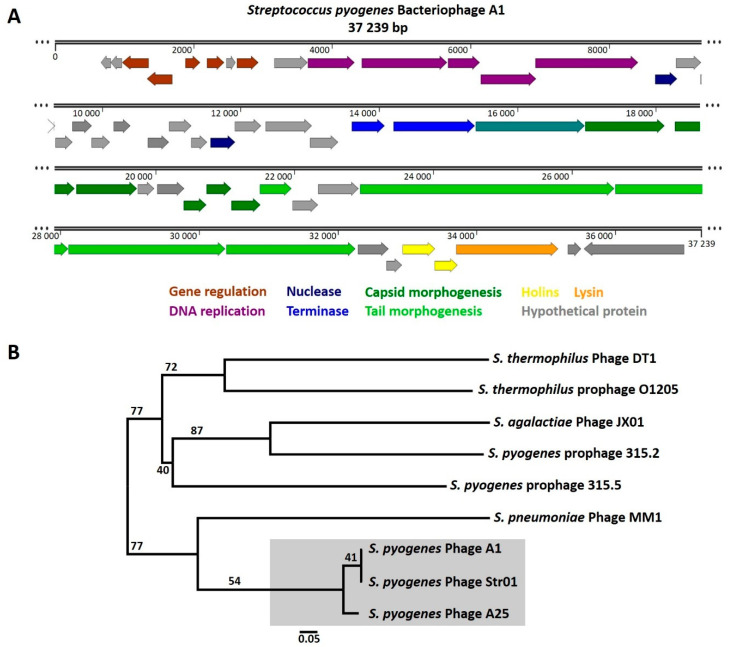
Genome sequence and phylogeny of *S. pyogenes* bacteriophage A1. (**A**) The Phage A1 genome is a 37,239 bp long circular dsDNA genome. Fifty-two gene products (Gps) were identiFigure 23. were encoding hypothetical proteins (gray). The genome follows the modular organization observed in other streptococcal phages, consisting of genes involved in regulation (brown), DNA replication (purple), nucleases (marine blue), DNA packaging (blue), portal protein (turquoise), capsid structure (dark green), tail morphogenesis (light green), holins (yellow) and a lysin (orange); (**B**) Whole genome-based phylogenetic tree of Phage A1 among streptococcal phages. The branch lengths are scaled in terms of the GBDP distance formula D_0_. Branch support was inferred from 100 pseudo-bootstrap replicates each.

To obtain a more complete understanding of the molecular basis of the Phage A1 biology and its interaction with *S. pyogenes*, we first sequenced the genome of Phage A1. We determined that the Phage A1 genome is a circular dsDNA genome of 37,239 base pairs with a GC content of 37.9% (Figure 2A). In total, 52 putative gene products (Gps) were predicted, of which 23 encode hypothetical proteins that could not be identified by BlastP. The genomic organization of Phage A1 includes modules dedicated to gene regulation, DNA replication, DNA restriction, genome packaging, phage morphology and bacterial lysis, which is often observed for the *Siphoviridae* family members. The genome does not encode a hyaluronidase and no known virulence genes were identified. About half of the Phage A1 genome is dedicated to DNA packaging, phage structure and host cell lysis. The lysis module contains two predicted holin genes in addition to a peptidoglycan hydrolase lysin. The other half of the Phage A1 genome consists of regions dedicated to gene regulation and DNA replication; however, the majority of predicted Gps encode hypothetical proteins. Manual inspection of these hypothetical proteins resulted in some interesting observations. For instance, the Gp located downstream of the Gp for lysin (Gp51) showed 51% identity to a prophage gene involved in external DNA acquisition inhibition called paratox. Furthermore, a large proportion of hypothetical proteins are conserved proteins with domains of unknown function (DUF) commonly detected in phages, which were previously shown to be a potential source of anti-host defense mechanisms [42]. Intriguingly, *gp6* in the Phage A1 genome harbors almost 99% identity to a putative DNA-binding antitoxin component found in *S. pyogenes* strain GA40634. Lastly, we identified a putative site-specific integrase (Gp52) and a lysogeny control module in the genome of Phage A1. The integrase gene is identical to the integrase of Phage Str01 but is unrelated to integrase genes found in *S. pyogenes* prophages. The most closely related integrase gene was predicted in *Streptococcus bovimastitidis* with an identity of almost 83% (Table 3).

Since we identified a putative site-specific integrase along a lysogeny control module in the genome of Phage A1, we hypothesized that Phage A1 could be a temperate phage instead of a strictly lytic phage. To achieve a global overview of the relatedness of Phage A1 to both Phage A25 and Phage Str01, but also to phages of other streptococcal species and prophages present in *S. pyogenes*, we investigated the phylogenetic relatedness among streptococcal phages. We included sequenced lytic and temperate phages of *S. pyogenes*, *S. pneumoniae*, *Streptococcus agalactiae*, *Streptococcus thermophilus*, *S. suis* and *Streptococcus dysgalactiae* in addition to annotated prophages found in various M-type *S. pyogenes* strains. Initial alignments of the Phage A1 genome to lytic Phage A25 and temperate Phage Str01 already suggested that Phage A1 is highly similar to Phage A25 and Phage Str01. However, the phylogenetic analysis based on whole phage genomes revealed that Phage A1, Phage A25 and Phage Str01 form a separate branch in the generated phylogenetic tree (Figure 2B). *S. pneumoniae* Phage MM1 is the most closely related phage to these three phages with *S. pyogenes* prophages, *S. suis* and *S. dysgalactiae* phages being the most distantly related (Figure S1). However, temperate Phage Str01 is the most similar phage to Phage A1, further corroborating the possibility that Phage A1 is a temperate phage and not a strictly lytic phage.

### 3.3. Phage A1 Is Potentially Temperate and Phage A1 Survivors Acquire Phage Resistance

**Figure 3:**
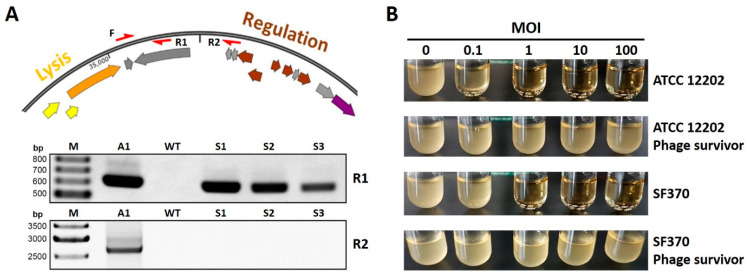
Phage A1 is likely a temperate phage, and Phage A1 survivors become resistant to subsequent infections. (**A**, top) Genomic DNA was screened by PCR for phage integration with primers specific to Phage A1 (F + R1) and primers that should not amplify an amplicon if the Phage DNA is integrated into the genome of S. pyogenes (F + R2) (F: forward, R: reverse); (A, bottom) Agarose gel showing PCR amplicons of both combinations F + R1 (up) and F + R2 (down) in S. pyogenes ATCC 12202. A1: Phage A1 genome, WT: wild type S. pyogenes ATCC 12202, S1: phage survivor 1, Scheme 2. phage survivor 2 and S3: phage survivor 3; (**B**) Representative pictures of *S. pyogenes* ATCC 12202 and SF370, and their phage-surviving strains after 4 h of infection at MOIs 0.1, 1, 10 and 100 compared to uninfected growth (MOI 0). Each image represents WT strains and one out of three phage-surviving clones that were infected in biological triplicates.

To test whether Phage A1 could be a temperate phage, we assessed its ability to integrate into the genome of *S. pyogenes* strains ATCC 12202 and SF370. Cultures in mid-logarithmic growth phase were infected with Phage A1, after which we spun down cleared lysates and plated pellets on blood agar plates to check for viable clones. To omit the possibility of picking up contaminating phage DNA, single colonies were selected and passaged twice before testing for the presence of Phage A1 products by PCR. The genomic DNA of phage-surviving colonies was subjected to PCR to screen for phage integration with primers specific to Phage A1 and primers that should not yield a product when the Phage DNA is integrated into the host genome (Figure 3A top). We could amplify a product from the genomic DNA of 6 of 14 SF370 and 13 of 15 ATCC 12202-surviving colonies when we screened for the Phage A1 integrase gene (Figure 3A bottom). In contrast, no amplicons could be detected when the PCR covered the circularization junction of the Phage A1 genome (Figure 3A), confirming that the Phage A1 genome is present in the isolated *S. pyogenes* genomic DNA.

Having established that Phage A1 DNA is present in *S. pyogenes* after infection, we investigated whether phage-surviving *S. pyogenes* clones become resistant to Phage A1 infection. We infected three Phage A1-surviving clones and their WT *S. pyogenes* strains with Phage A1 at MOIs ranging from 0.1 to 100. Phage A1-surviving clones of both ATCC 12202 and SF370 were able to grow even when infected at an MOI of 100. In contrast, WT strains were rapidly cleared when infected at MOI 0.1 for ATCC 12202 or MOI 1 for SF370 (Figure 3B).

### 3.4. Phage A1-Surviving Strains Do Not Alter the Host Immune Response or Tolerance to Antibiotics

Phage lysogeny has the potential to alter phenotypes of their hosts. Therefore, we investigated whether Phage A1 survivors behave differently than their WT counterparts. First, we determined growth characteristics to investigate the fitness of survivor strains. We observed a prolonged lag phase for all three tested ATCC 12202 phage-surviving clones, and these strains did not reach equal ODs when entering stationary growth phase. In contrast, each isolated phage-surviving SF370 strain behaved differently when compared to each other or to WT SF370. Surviving clone 1 grew equally during lag phase and early log phase but entered stationary phase earlier. Surviving clone 2 grew equal to WT but reached a higher OD. Surviving clone 3 showed a prolonged lag phase, similar to phage survivor ATCC 12202 strains (Figure S2).

**Figure 4:**
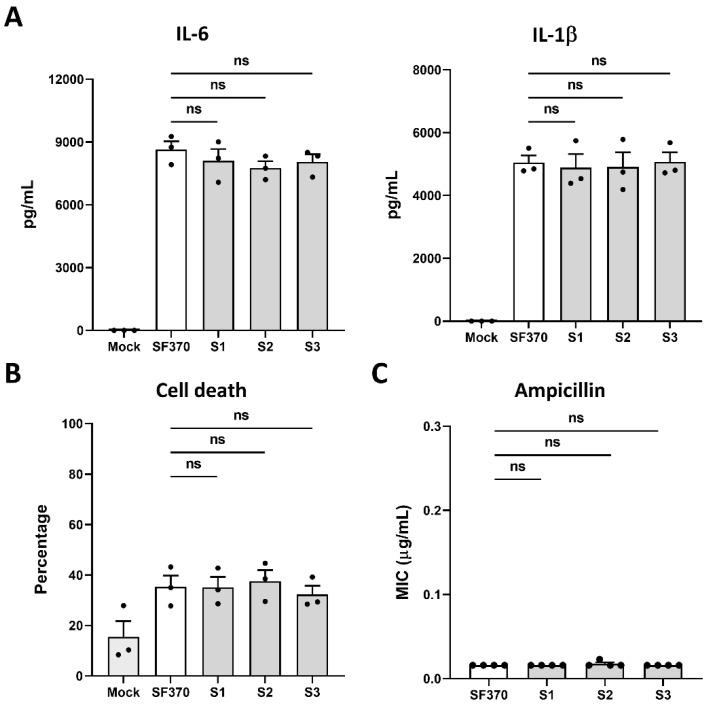
Phage A1-surviving strains do not influence host immune responses or antibiotic tolerance compared to wild type. (**A**) Overnight cytokine production by BLaER1 cells after infection with WT *S. pyogenes* SF370 and phage-surviving strains. (**B**) The percentage of BLaER1 cell death after infection with Phage A1 survivors or WT *S. pyogenes* SF370 is shown. (**A**,**B**) Mock: buffer control, S1: phage survivor 1, S2: phage survivor 2 and S3: phage survivor 3; (**C**) Ampicillin susceptibility profiles of *S. pyogenes* SF370 and survivor strains are shown. S1: phage survivor 1, S2: phage survivor 2 and S3: phage survivor 3; (A–C) Pooled data from three or four independent experiments are shown. Statistical significance between groups was determined by one-way ANOVA tests.

Since the antibiotic treatment failure of severe *S. pyogenes*-associated disease has been reported and phage therapy could provide an alternative, we examined whether the pathogenicity of Phage A1 survivors was affected in comparison to WT *S. pyogenes*. In the body, after *S. pyogenes* invades soft tissue and establishes systemic infection, it encounters various types of immune cells. Here, monocytes fulfil crucial functions during bacterial infection such as phagocytosis, antigen presentation and cytokine production [43]. We used BLaER1 cells to represent the encountered monocytes during systemic infection. BLaER1 are immortalized, genetically modified B-cells from a leukemia patient that stably express an inducible C/EBPα transgene. Induction of the transcription factor C/EBPα leads to the irreversible differentiation of BLaER1 cells into monocyte-like cells which recapitulate monocytic functions [41]. First, we investigated cytokine production by BLaER1 cells after infection with WT *S. pyogenes* SF370 and phage-surviving strains. We observed that infection with both WT and Phage A1 survivors results in a clear proinflammatory response, but found no difference in the amount of IL-6 or IL-1β produced by cells infected with WT SF370 compared to Phage A1 lysogen-infected cells (Figure 4A). Continuing to test the pathogenicity of Phage A1 survivors, we measured LDH release to determine the cytotoxic ability upon infection. In agreement with the cytokine profiles, we did not detect significant differences in released LDH in culture medium after infecting BLaER1 cells with WT SF370 or Phage A1-surviving strains (Figure 4B).

As the tolerability of *S. pyogenes* towards antibiotics can be influenced upon phage lysogenization or chromosomal insertion of prophage-associated genetic elements [44], we investigated the antimicrobial resistance profile against the commonly used antibiotic ampicillin. Here, we observed that Phage A1-surviving strains are not enhanced in their ability to grow in the presence of ampicillin, showing no increase in MIC (Figure 4C).

### 3.5. CRISPR-Cas-Mediated Spacer Acquisition Is Increased in the Absence of a Capsule

**Figure 5:**
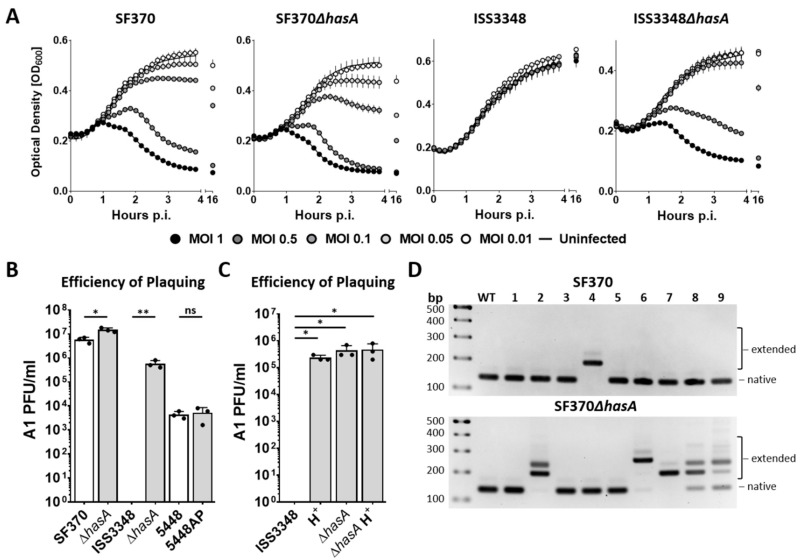
Impaired capsule-based phage resistance results in more detectable CRISPR-Cas-mediated spacer acquisition. (**A**) Lysis profiles of Phage A1 on *S. pyogenes* SF370, ISS3348, and their isogenic *ΔhasA* strains. Each curve shows mean values ± SD of one out of three experiments in triplicates; (**B**) Phage A1 efficiency of plaquing (EOP) on various M1 serotype *S. pyogenes*, and their isogenic *ΔhasA* or CovS mutant strain; (**C**) Phage A1 EOP on *S. pyogenes* ISS3348 and ISS3348*ΔhasA* grown in the absence or presence of hyaluronidase (H^+^); (**B**,**C**) Results are presented as mean values ± SD from three independent experiments. *p* < 0.05 determined by paired t tests was considered statistically significant. * *p* < 0.05, ** *p* < 0.01, NS = not significant; (**D**) Representative agarose gels showing amplified CRISPR arrays of WT *S. pyogenes* SF370 (up), SF370*ΔhasA* (down), and 9 surviving colonies (1–9) for both strains after Phage A1 infection.

It is well known that *S. pyogenes* produces an HA capsule, which has been shown to play an important factor in the defense against phage infection or in immune evasion [16,17,18]. However, capsule production differs greatly among strains and under different growth conditions [45]. To examine the importance of the capsule in the defense against Phage A1 infection, we investigated the lysis profile of Phage A1 on commonly studied *S. pyogenes* SF370 and clinical isolate *S. pyogenes* ISS3348. The latter strain contains a naturally occurring mutation in *covRS* resulting in enhanced capsule production. Additionally, *hasA* KO mutants of SF370 and ISS3348 were tested to identify the role of the capsule during Phage A1 infection. *S. pyogenes* SF370 showed rapid lysis after Phage A1 infection at MOI 1 and MOI 0.5, but was delayed at MOI 0.1. A minor decrease in OD was observed after overnight infection at MOI 0.05 and no lysis was observed at lower MOIs than 0.05. The lytic phenotype on SF370*ΔhasA* was increased compared to WT SF370, with accelerated lysis occurring up to MOI 0.05. Lysis at MOI 0.01 could not be detected. In contrast, ISS3348 showed a completely resistant phenotype to Phage A1 infection with no lysis occurring at the tested MOIs. However, the lysis profile of Phage A1 on ISS3348*ΔhasA* was similar to that of SF370, showing that ISS3348 becomes susceptible to Phage A1 infection in the absence of its capsule (Figure 5A).

Since CovRS regulates the expression of up to 15% of the *S. pyogenes* genome—including the *hasABC* operon—[46] it is not known whether an altered infection of ISS3348 would be a direct consequence of the mutated *covRS* resulting in the thickened capsule or other background-related factors compared to SF370. Therefore, we included *S. pyogenes* 5448 and 5448AP, which are identical except for an inactivated CovRS system in 5448AP [34]. We assessed plaquing efficiency to confirm the ability of Phage A1 to infect the aforementioned M1 serotype *S. pyogenes* strains and their mutants. Here, we confirmed that Phage A1 infectivity was slightly increased on SF370*ΔhasA* compared to SF370. Additionally, Phage A1 was unable to form PFUs on strain ISS3348 but we could detect PFUs on ISS3348*ΔhasA*, which were up to one order of magnitude less compared to WT SF370. The infection of strain 5448 was 2.5 to 3 orders of magnitude lower compared to SF370, but PFU counts showed a similar infection efficiency as on 5448AP (Figure 5B). To further verify that the increased susceptibility of the *hasA* KO mutants is due to the absence of the capsule, we included hyaluronidase in the growth medium of WT *S. pyogenes* strains to prevent capsule formation and repeated EOP assays. Here, we confirmed that the infection efficiency of Phage A1 was increased by around one order of magnitude when *S. pyogenes* was grown in hyaluronidase-containing media, similar to the difference between WT SF370 and SF370*ΔhasA* without hyaluronidase in growth media (Figure S3A). Strikingly, WT ISS3348 shifted from a completely resistant phenotype to a susceptible phenotype when grown in hyaluronidase-containing media. The infection of WT ISS3348 by Phage A1 was similar to the infection of ISS3348*ΔhasA*. The addition of hyaluronidase in the growth media had no additional effect on the infection of ISS3348*ΔhasA*, confirming that the *S. pyogenes* capsule is pivotal in the defense against phage infection (Figure 5C).

Although Phage A1 infectivity was increased for the *hasA* KO mutants, we observed that phage-surviving colonies in the absence of a capsule could still be recovered after overnight growth. Consequently, we investigated intracellular mechanisms by which *S. pyogenes* could resist phage infection. We established a spacer acquisition assay adapted from a previous study [47] to test whether the type II-A CRISPR-Cas system of *S. pyogenes* could acquire new spacers deriving from Phage A1. *S. pyogenes* SF370 and SF370*ΔhasA* were infected at MOI 1 after which the cleared culture was probed for viable colonies on blood plates. Surviving colonies were screened for spacer acquisition by PCR after excluding the presence of Phage A1 DNA in the same strain. We detected the acquisition of a new spacer in 2 out of 27 tested surviving single colonies of WT SF370. Interestingly, we observed spacer acquisition in 12 out of the 27 tested single colonies in the *hasA* KO strain. Additionally, multiple acquired spacers could be amplified from the genome of SF370*ΔhasA*, suggesting that spacer acquisition is increased in the absence of a capsule (Figure 5D). Sequencing of acquisition events confirmed that the spacers derive from the Phage A1 genome. The acquired spacers in WT SF370 targeted structural proteins encoding the major capsid protein (Gp33) and the minor structural tail protein (Gp44) in the genome of Phage A1. In addition to Gps 33 and 44, the putative transcriptional regulator (Gp08), DNA primase (Gp14), structural tail protein (Gp45) and the lysin (Gp50) were targeted in SF370*ΔhasA* (Figure S3B).

### 3.6. Membrane Vesicles of S. pyogenes Bind Phages and Limit Phage Infection

**Figure 6:**
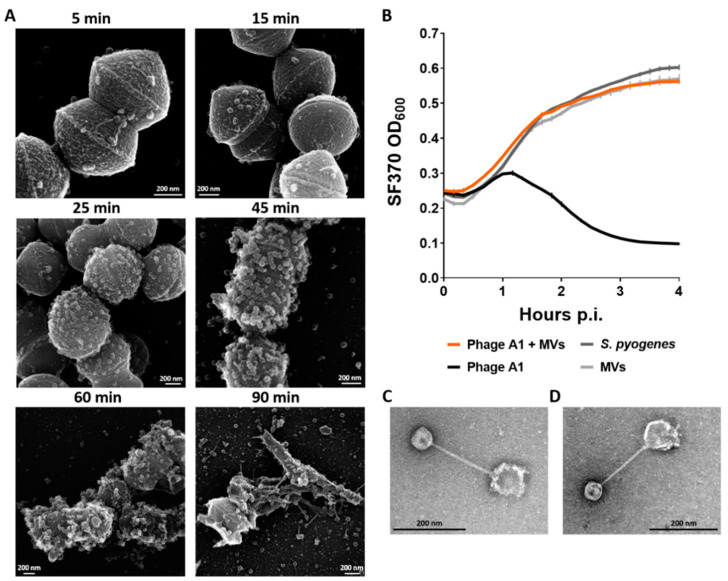
*S. pyogenes*-derived membrane vesicles inhibit phage infection. (**A**) Representative SEM micrographs of *S. pyogenes* SF370-infected with Phage A1 at MOI 10 are shown after 5, 15, 25, 45, 60 and 90 min of coincubation; (**B**) Growth curves of WT SF370 after Phage A1 infection that was either pre-incubated with membrane vesicles (MVs) or left untreated. Phage:*S. pyogenes*:MV ratio of 1:1:1000 is shown. Mean values ± SD from one out of three independent experiments in Table 1. bound to a *S. pyogenes* SF370-derived MV; (**D**) TEM micrograph of a *S. pyogenes* ISS3348-derived MV bound to Phage A1; (**C**,**D**) Representative images are shown for at least three sample preparations.

Assessing the lysogeny and CRISPR spacer acquisition of *S. pyogenes* SF370 showed that the type II CRISPR-Cas system integrated new spacers into its CRISPR array and that the strain was less efficiently lysogenized by Phage A1. In contrast, nearly all phage-surviving *S. pyogenes* ATCC 12202 colonies contained detectable Phage A1 DNA. Furthermore, the capsule of ISS3348 seems to be the critical determinant in the defense against phage infection. Therefore, we hypothesized that mechanisms by which *S. pyogenes* survives phage infection can vary among strains and are not restricted to lysogeny, the capsule or CRISPR-Cas immunity. Another strategy by which bacteria can limit phage infection is by the increased production of MVs, which could potentially serve as a decoy for phages [48]. To explore the potential role of *S. pyogenes*-derived MVs in the defense against Phage A1 infection, we followed phage infection over time with SEM imaging after infecting *S. pyogenes* SF370 with Phage A1 at an MOI of 10. Here, we observed that phages are bound to their host after 5 min of incubation, and disturbed bacterial membranes and potential MV formation became visible after 25 min of infection. MV production was clearly observed after 45 min of infection. The first lysed cells appeared between 45 and 60 min of infection. Additionally, most *S. pyogenes* cells were lysed after 90 min (Figure 6A).

To evaluate whether increased amounts of MVs produced by *S. pyogenes* are able to limit phage predation, we incubated purified MVs of *S. pyogenes* SF370 with Phage A1 before infecting *S. pyogenes* SF370. Indeed, we observed the inhibition of phage infection with phage:*S. pyogenes*:MV ratios of 1:1:1000 (Figure 6B). In contrast, we did not observe the inhibition of phage infection when we infected *S. pyogenes* at higher phage to MV ratios in favor of phage amounts (not shown), suggesting a concentration-dependent inhibitory effect. To examine whether the observed neutralizing effect is due to the direct binding of the phage to MVs, we visualized coincubated Phage A1 and purified *S. pyogenes* SF370-derived MVs with TEM. Here, we observed that phages were bound to MVs after 15 min with clear individual contact between the phage tail and the MVs (Figure 6C). To investigate whether the binding of Phage A1 to MVs is a general phenomenon among *S. pyogenes* strains, we purified MVs from clinical isolate ISS3348 and tested the interaction between Phage A1 and ISS3348-derived MVs. We observed that phages were bound to MVs of ISS3348 after coincubation, and interaction between phage and MVs was observed (Figure 6D).

## 4. Discussion

In this study, we describe a previously uncharacterized *S. pyogenes* phage in detail. We show that Phage A1 is a putative temperate phage and that its DNA can be detected in *S. pyogenes* genomes, resulting in strains that are resistant to subsequent infections. Importantly, these Phage A1-survivor strains do not develop ampicillin tolerance or become more virulent than their WT counterpart.

To date, the best-studied phage of *S. pyogenes*—that is not a prophage—is Phage A25. Electron micrographs showed that Phage A25 belongs to the *Siphoviridae* [26], and although minor differences exist in some phage dimensions, the morphology of Phage A1 is highly similar to that of Phage A25. The estimated burst size of Phage A25 was determined at 12 to 32 PFU per bacterial cell depending on the host strain that was investigated [27,28]. Similarly, we observed an average burst of 31 PFUs per infected cell for Phage A1 when propagated on *S. pyogenes* ATCC 12202. However, this number could differ when tested on different *S. pyogenes* strains since the lysis profile also showed minor differences in the infection cycle between *S. pyogenes* strains ATCC 12202 and SF370.

Streptococcal phages typically feature a modular genome organization with a clear distinction between regions dedicated to phage structure and host cell modulation [49]. Recently, two *S. pyogenes* phage genomes were sequenced where this modular genetic arrangement was observed as well [30,50]. The Phage A1 genome was highly similar to temperate Phage Str01 and virulent Phage A25, with Phage Str01 being its closest relative. Compared to Phage Str01, Phage A1 accommodates 37 more nucleotides in a non-coding region dedicated to gene regulation and 132 nucleotides more in a hypothetical protein produced by *g23* that were present in the Phage A25 genome. Furthermore, we identified two single nucleotide differences, one of which was a synonymous substitution in the DNA primase. The other difference, however, was a non-synonymous mutation in the DUF1366-protein changing a valine to isoleucine in comparison to Str01. Besides the missing integrase gene and surrounding genetic regions in Phage A25, multiple (non-) synonymous mutations were present in several structural proteins but also in hypothetical proteins. Two amino acid changes were detected in the C-terminus of the class I Holin, which were not present in the Phage Str01 genome. One of the Phage A1 genes (*g52*) shared identity with a recombinase family protein found in *S. bovimastitidis* and is identical to the site-specific integrase gene of Phage Str01, which is noteworthy since Phage A1 is historically implicated to be a strictly lytic phage [18,24]. Here, we observe that the integrase gene locates in the vicinity of the gene regulation module, which is expressed early during infection (data not shown). Early expressed genes may be involved in lysogenic–lytic lifecycle decisions, and local transcript levels could therefore determine which replication cycle the phage enters, e.g., high concentrations of integrase transcripts promoted a lysogenic state of mycobacteriophages upon infection [51]. Phage A25 escaped from lysogeny by the deletion of its integrase [30], and, based on the genomic similarity and differences between Phage Str01, Phage A25 and Phage A1, it is tempting to speculate that Phage A1 served as an intermediate phage in the conversion of Phage A25 from a temperate to a strictly lytic phage.

PCR analysis showed that Phage A1 DNA can be amplified from the genome of nearly all *S. pyogenes* ATCC 12202- and some SF370-surviving colonies, indicating that Phage A1 could be a temperate phage. Furthermore, surviving Phage A1 infection in both parental *S. pyogenes* strains resulted in complete resistance against subsequent phage infections, most likely by providing superinfection immunity. Previous hints towards superinfection immunity against Phage A25 were found by McCullor and colleagues, where they observed that *S. pyogenes* strains containing high homology prophages to Phage A25 were resistant to infection [30]. The mechanism of resistance was experimentally confirmed by complementing the *cI*-like repressor and regulation module of an *S. pyogenes* prophage—representing the deleted lysogeny module that is absent in Phage A25 but present in Phage A1—in multiple strains that were susceptible to Phage A25 infection. As a result, complemented strains converted into phage-resistant strains through the expression of the *cI*-like repressor, which prevents the transcription of invading homologous phages and terminates phage replication [30].

*S. pyogenes* is one of the most clinically relevant human pathogens causing over 500,000 deaths each year [6]. Prophages in the genome of *S. pyogenes* have been shown to be a major contributor to virulence of this pathogen by transmitting virulence factors that altered its pathogenic behavior [52,53,54]. We did not identify known virulence genes in the genome of Phage A1, but previous studies have shown that lysogeny can significantly influence the host cell by altering the bacterial surface or transduce virulence factors to other host cells [55]. Our lab and others previously described the temperate phage-mediated transfer of the streptococcal superantigen genes *ssa* [56] and *speA* [57] among clinical *S. pyogenes* isolates. Nevertheless, phage therapy holds great potential to treat complicated infections where antibiotics are ineffective. Because temperate phages have the ability to integrate into host genomes and mediate the transfer of potential virulence genes between bacteria, strictly lytic phages are preferred subjects for therapeutic purposes [58]. In this study, however, we did not observe an increased antibacterial resistance phenotype or pathogenicity of Phage A1 survivors compared to WT bacteria, which holds promise for the development of Phage A1 or Phage A1-derived products for therapeutic uses. For instance, Phage A1 could be engineered towards a lytic phage by the removal of the putative integrase, or to disturb intracellular processes that are important for bacterial survival after lysogenization [59]. However, Phage A1 survivors displayed a superinfection immunity phenotype and were insensitive to subsequent phage infections. Additionally, early studies have described increased virulence of phage-surviving *S. pyogenes*, showing that phage survivors were enhanced in their ability to kill mice [24], although a real estimation of virulence is difficult due to discrepancies between our infection model and mouse models used in the 1950s. In conclusion, a cautious approach towards temperate phages in phage therapy should be kept in mind and we suggest the development of phage endolysins from streptococcal phages for their antibacterial potential over the therapeutic use of Phage A1 itself, to support the treatment of severe streptococcal disease where antibiotics failed to resolve the infection [60].

The hyaluronic acid capsule is a major virulence determinant for *S. pyogenes* and highly encapsulated *S. pyogenes* strains are associated with more severe disease and increased resistance to neutrophil-mediated killing [16,17]. Moreover, the removal of the HA capsule increased the phage sensitivity of *S. pyogenes* [18,30]. Here, we were able to confirm the critical role of the capsule in phage defense. We observed that Phage A1 infects SF370 as efficiently as its replication strain ATCC 12202 but did not propagate in *S. pyogenes* ISS3348. *S. pyogenes* ISS3348 is a clinical isolate with a mutation in the two-component regulatory system CovRS. Mutations in CovRS have been shown to result in highly encapsulated *S. pyogenes* strains and could explain the observation that ISS3348 is resistant to phage infection since increased capsule production blocks phage adsorption [14]. In agreement, Phage A1 infection of ISS3348 was observed when *hasA* was deleted from the genome or when the capsule was removed by enzyme treatment in the growth media, confirming that the capsule is a direct cause of impenetrability. However, we observed equal infection efficiencies of Phage A1 on *S. pyogenes* strains 5448 and 5448AP, indicating that background-related factors in addition to the thickness of the capsule are involved in phage resistance and can differ between strains.

Interestingly, we observed that the type II-A CRISPR-Cas system of SF370*ΔhasA* showed elevated spacer acquisition events compared to WT spacer acquisition levels. Previous studies showed that CRISPR loci were among the most highly transcribed regions in bacterial cells during normal growth [61,62], suggesting that the high-level expression of CRISPR-Cas systems does not result in fitness defects and is advantageous for survival, at least in some environments. In agreement, no differential expression of the type II-C CRISPR-Cas genes in *C. jejuni* was observed upon phage infection [63]. Therefore, we hypothesized that native expression levels of the SF370 CRISPR-Cas system are sufficient for phage defense but the increased entry of Phage A1 into cells without a capsule and the involved dynamics hereof resulted in increased phage recognition by the CRISPR-Cas system. Until now, the CRISPR-Cas system of *S. pyogenes* has not been investigated in its native host due to the previous lack of infectious elements to challenge *S. pyogenes*. Although the type II-A CRISPR-Cas system has been studied extensively in a heterologous host [47,64], it would be most important to study the type II-A CRISPR-Cas system in its native settings. For instance, host-specific factors are absent in previous experimental set-ups and could play important parts in CRISPR immunity, as shown for other organisms [65]. Based on our results, Phage A1 and *S. pyogenes* SF370*ΔhasA* provide an ideal system to study the CRISPR-Cas system to gain a deeper understanding of CRISPR-Cas biology in its original host, which ultimately could lead to the optimization of CRISPR-Cas9-based tools for biotechnological purposes.

Another characteristic of *S. pyogenes* strains that acquired *covRS* mutations is the hyper production of MVs [35]. MVs are naturally secreted by cells of all kingdoms of life and exhibit a large diversity of biological roles [66]. Because the surface of MVs is highly similar to that of their host cells, MVs can serve as a decoy for phages and limit phage infection [66,67]. In this study, we observed highly disturbed bacterial membranes and MV formation during the course of infection. Additionally, incubating purified MVs of *S. pyogenes* SF370 with Phage A1 before the infection of *S. pyogenes* SF370 inhibited phage infection. We could further show that the observed neutralizing effect is due to direct binding of the phage to MVs that could also be applied to other strains of *S. pyogenes*. Other studies have reported similar observations. *Escherichia coli* outer membrane vesicles (OMVs) were shown to neutralize bacteriophage T4 by the formation of complexes between phage and OMVs [48]. Moreover, the OMVs of *Vibrio cholerae* reduced phage infection in a dose- and receptor-dependent manner [67], providing evidence that MVs act as a first line of defense against phage predation that can be applied to a large variety of bacterial species. However, the extent to which MVs contribute towards the phage resistance of *S. pyogenes* in natural habitats is less understood since the concentrations of MVs that naturally accumulate during infection are unknown. Additionally, mutants of *S. pyogenes* that are unable to produce MVs have not been obtained to date, which makes it impossible to quantify the role that MVs play during infection. Nevertheless, it is reasonable to assume that MVs can be protective of phage infection when a desirable ratio of MVs and phages has accumulated in its micro-environment.

In summary, we propose a multistage interaction model between *S. pyogenes* and Phage A1. 

MVs produced by *S. pyogenes* are able to scavenge phage particles from the environment and reduce the infection pressure. When phages reach the cell surface of *S. pyogenes*, they have to overcome a hyaluronic acid capsule to bind to their receptor. When a phage manages to bind its receptor and injects its DNA into *S. pyogenes*, new phages are produced in the majority of infected cells, which are released during cell burst. When the capsule is absent or reduced in thickness, phage infectivity is increased and intracellular anti-phage defense systems—such as CRISPR-Cas—become more prominent. In a minor fraction of infected cells, the Phage A1 genome becomes present in the *S. pyogenes* genome enabling the survival of the bacterial host. However, Phage A1-surviving clones do not become more virulent than their WT counterpart in vitro or develop resistance towards ampicillin. In conclusion, this work provides novel insight into survival strategies utilized by *S. pyogenes* to combat phage predation and describes a means to study the type II-A CRISPR-Cas system in its native host. Lastly, we propose that Phage A1 gene products—such as the endolysin—have the potential to be harnessed for the development of anti-bacterial agents. 

## Figures and Tables

**Table 1:** *S. pyogenes* strains used in this study.

Strain	Description	Origin
ATCC 12202	*Streptococcus pyogenes* Rosenbach (ATCC^®^ 12202™)	ATCC database
SF370	*Streptococcus pyogenes* Rosenbach (ATCC^®^ 700294™)	Ferretti et al. 2001 [31] and Abbot et al. 2007 [32]
SF370*ΔhasA*	SF370 with complete deletion of the *hasA* coding sequence	Abbot et al. 2007 [32]
5448	Wild-type clinical STSS/Necrotizing Fasciitis isolate	M. Walker, University of Queensland, Australia [33]
5448AP	Animal passaged variant of 5448 containing a 1-bp insertion in *covS*	M. Walker, University of Queensland, Australia [34]
ISS3348	Clinical isolate; 30-bp deletion in *covS*	G. Teti, University of Messina, Italy
3348*ΔhasA*	ISS3348 with complete deletion of the *hasA* coding sequence	Resch et al. 2016 [35]

**Table 2:** Phage A1 characteristics.

Phage Parameter	Bacteriophage A1 (nm ± SD *)
Head	60 ± 2
Tail length	187 ± 4
Tail width	10 ± 1
Baseplate width	16 ± 2
Total length	249 ± 3

* Standard Deviation.

**Table 3:** Phage A1 genome annotation.

Genomic Location (Gp Number)	Identified Protein (Species)	Direction	Accession Number	E Value	Identity %
667..801 (Gp01)	Hypothetical phage protein (*S. pyogenes* Phage 10270.2)	<=	ABF33863.1	1e−21	100
808..960 (Gp02)	Hypothetical protein (*S. pyogenes*)	<=	WP_011054825.1	5e−27	100
971..1348 (Gp03)	ImmA/IrrE family metallo-endopeptidase (*S. pyogenes*)	<=	WP_011054824.1	2e−86	100
1332..1691 (Gp04)	XRE family transcriptional regulator *(S. pyogenes* Phage Str01)	<=	APZ81910.1	2e−79	100
1881..2099 (Gp05)	Cro family anti-repressor (*S. pyogenes* Phage 10270.2)	=>	ABF33867.1	2e−44	100
2194..2445 (Gp06)	Putative DNA-binding antitoxin (*S. pyogenes* GA40634)	=>	EPZ46884.1	4e−46	98.65
2476..2610 (Gp07)	Hypothetical protein (*S. pyogenes* Phage 10270.2)	=>	ABF33611.1	5e−24	100
2626..2940 (Gp08)	Helix-turn-helix transcriptional regulator (*S. pyogenes*)	=>	WP_011528544.1	2e−69	100
3167..3649 (Gp09)	Hypothetical Gp157 family protein (*S. pyogenes*)	=>	WP_011528545.1	2e−108	100
3650..4330 (Gp10)	AAA family ATPase (*S. pyogenes* MGAS1882)	=>	AFC68359.1	2e−164	99.56
4432..5661 (Gp11)	DEAD/DEAH box helicase (*Streptococcus sp*.)	=>	WP_011528546.1	0	100
5677..6135 (Gp12)	DUF669 domain-containing protein (*Streptococcus sp*.)	=>	WP_002995969.1	2e−107	100
6147..6950 (Gp13)	Bifunctional DNA primase/polymerase (*S. pyogenes*)	=>	WP_011528547.1	0	99.25
6940..8421 (Gp14)	DNA primase (*S. pyogenes*)	=>	WP_020905118.1	0	99.8
8666..8986 (Gp15)	VRR-NUC domain-containing protein (*S. pyogenes*)	=>	WP_002995960.1	7e−71	100
8970..9326 (Gp16)	Hypothetical protein (*S. pyogenes*)	=>	WP_011018138.1	4e−79	100
9323..9574 (Gp17)	Hypothetical protein (*S. pyogenes*)	=>	WP_011528549.1	4e−54	100
9568..9852 (Gp18)	Hypothetical DUF3310-containing protein (*S. pyogenes*)	=>	WP_011017568.1	5e−64	100
9849..10,118 (Gp19)	Hypothetical protein (*S. dysgalactiae*)	=>	SUN67275.1	1e−58	98.88
10,170..10,409 (Gp20)	Hypothetical TIGR01671 family protein (*S. pyogenes* GA40634)	=>	ESA49192.1	6e−33	98.28
10,660..10,971 (Gp21)	Hypothetical DUF1372-containing protein (*S. dysgalactiae*)	=>	WP_081281150.1	9e−62	92.31
10,971..11,294 (Gp22)	Hypothetical protein *(S. pyogenes* Phage Str01)	=>	APZ81913.1	2e−70	100
11,287..11,517 (Gp23)	Hypothetical protein *(S. pyogenes* Phage A25)	=>	YP_009191526.1	2e−47	100
11,565..11,924 (Gp24)	Endodeoxyribonuclease RusA (*S. dysgalactiae*)	=>	WP_065359284.1	7e−81	97.48
11,917..12,297 (Gp25)	Hypothetical protein *(S. pyogenes* Phage Str01)	=>	APZ81908.1	7e−86	100
12,364..13,035 (Gp26)	Hypothetical DUF4417-containing protein (*S. dysgalactiae*)	=>	WP_155778398.1	3e−164	98.21
13,005..13,415 (Gp27)	Hypothetical protein *(S. pyogenes* Phage Str01)	=>	APZ81907.1	2e−87	100
13,608..14,087 (Gp28)	Terminase (small subunit) (*S. dysgalactiae*)	=>	WP_003058573.1	6e−111	100
14,209..15,387 (Gp29)	Terminase (large subunit) (*S. dysgalactiae*)	=>	WP_003058577.1	0	100
15,399..16,976 (Gp30)	Portal protein (*S. dysgalactiae*)	=>	WP_003058556.1	0	100
16,979..18,127 (Gp31)	Minor capsid protein (*S. dysgalactiae*)	=>	WP_003058578.1	0	100
18,274..18,837 (Gp32)	Scaffolding protein *(S. pyogenes* Phage A25)	=>	YP_009191535.1	2e−129	100
18,856..19,734 (Gp33)	Major capsid protein (*Streptococcus canis*)	=>	WP_164406187.1	0	97.26
19,745..19,981 (Gp34)	Hypothetical protein (*S. dysgalactiae*)	=>	WP_003058596.1	3e−46	100
20,025..20,417 (Gp35)	Hypothetical protein (*S. dysgalactiae*)	=>	WP_003058548.1	9e−91	100
20,407..20,733 (Gp36)	Minor capsid protein *(S. pyogenes* Phage Str01)	=>	APZ81883.1	8e−70	100
20,733..21,092 (Gp37)	Capsid protein *(S. pyogenes* Phage A25)	=>	YP_009191540.1	4e−82	100
21,092..21,511 (Gp38)	Capsid protein (*S. dysgalactiae*)	=>	WP_155783005.1	4e−96	100
21,504..21,962 (Gp39)	Major tail shaft protein *(S. pyogenes* Phage A25)	=>	YP_009191542.1	6e−107	100
21,977..22,345 (Gp40)	Hypothetical protein (*S. dysgalactiae*)	=>	WP_155783003.1	1e−81	100
22,345..22,929 (Gp41)	Hypothetical Gp15 protein (*Streptococcus equi* subsp. *zooepidemicus*)	=>	KIS17966.1	4e−88	68.39%
22,946..26,617 (Gp42)	Tail tape measure protein *(S. pyogenes* Phage Str01)	=>	APZ81886.1	0	100
26,626..28,116 (Gp43)	Tail endopeptidase *(S. pyogenes* Phage Str01)	=>	APZ81874.1	0	100
28,120..30,381 (Gp44)	Minor structural tail protein *(S. pyogenes* Phage A25)	=>	ALF02720.1	0	100
30,393..32,258 (Gp45)	Structural tail protein *(S. pyogenes* Phage A25)	=>	ALF02721.1	0	100
32,291..32,737 (Gp46)	Hypothetical DUF1366-containing protein (*S. dysgalactiae*)	=>	WP_003058584.1	5e−97	99.3
32,703..32,930 (Gp47)	Hypothetical protein *(S. pyogenes* Phage Str01)	=>	APZ81920.1	4e−45	100
32,938..33,405 (Gp48)	Class I Holin *(S. pyogenes* Phage A25)	=>	YP_009191551.1	2e−106	98.71
33,398..33,733 (Gp49)	Class III Holin (*S. dysgalactiae*)	=>	WP_003058565.1	6e−74	100
33,708..35,180 (Gp50)	Lysin *(S. pyogenes* Phage A25)	=>	YP_009191553.1	0	100
35,318..35,509 (Gp51)	Hypothetical Paratox protein (*S. pyogenes*)	=>	WP_136110174.1	2e−06	51.22
35,552..36,991 (Gp52)	Hypothetical Recombinase family protein (*S. bovimastitidis*)	<=	WP_071794279.1	0	82.88

## Data Availability

Not applicable.

## Supplementary Materials

The following are available online at https://www.mdpi.com/article/10.3390/v13040612/s1, Figure S1: Extended phylogeny of Phage A1 to streptococcal phages based on whole genomes; Figure S2: Growth curves of WT ATCC 12202 (left), WT SF370 (right) compared to three potential Phage A1 lysogens each; Figure S3: (A) Phage A1 plaquing on SF370 in the absence and presence of hyaluronidase in growth medium. Mean values ± SD from three independent experiments are shown. (B) Alignment of acquired spacer sequences in WT SF370 (top) or SF370*ΔhasA* (bottom) phage survivors to the Phage A1 genome highlighted in red on the dark yellow bar, marked by asterisks
