# Supplementary material for: Survival Strategies of Streptococcus pyogenes in Response to Phage Infection
Source: Viruses. 2021 Apr 2;13(4):612. doi: 10.3390/v13040612 (PMC8066415; doi:10.3390/v13040612)
Supplement: Supplementary file 1 [file viruses-13-00612-s001.pdf]

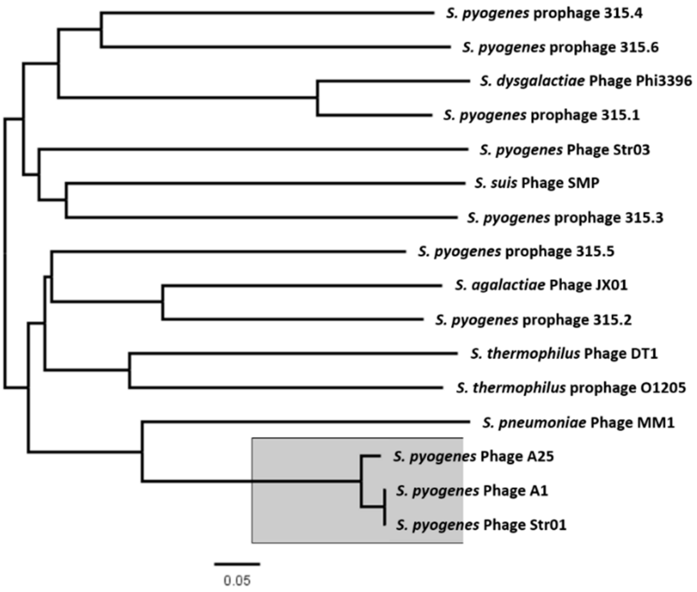

Figure 1. Extended phylogeny of Phage A1 to streptococcal phages based on whole genomes. The branch lengths are scaled in terms of the GBDP distance formula D0. Branch support was inferred from 100 pseudo-bootstrap replicates each.

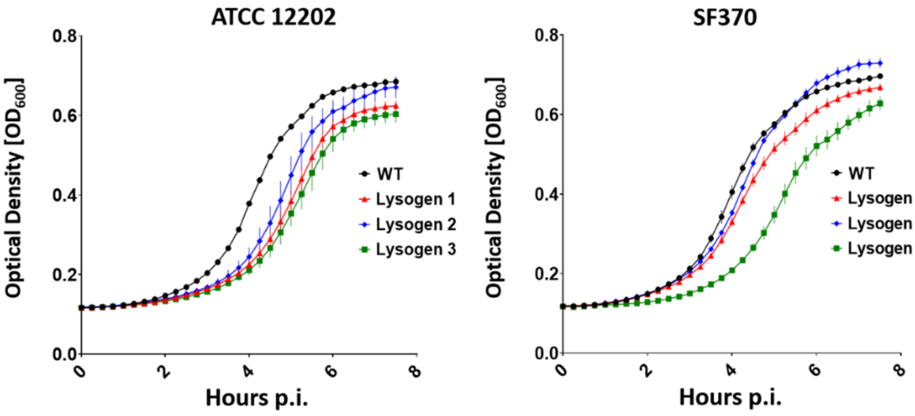

Figure 2. Growth curves of WT ATCC 12202 (left), WT SF370 (right) compared to three Phage A1 lysogens each. Mean values ± SD from one out of two independent experiments in triplicates are shown.

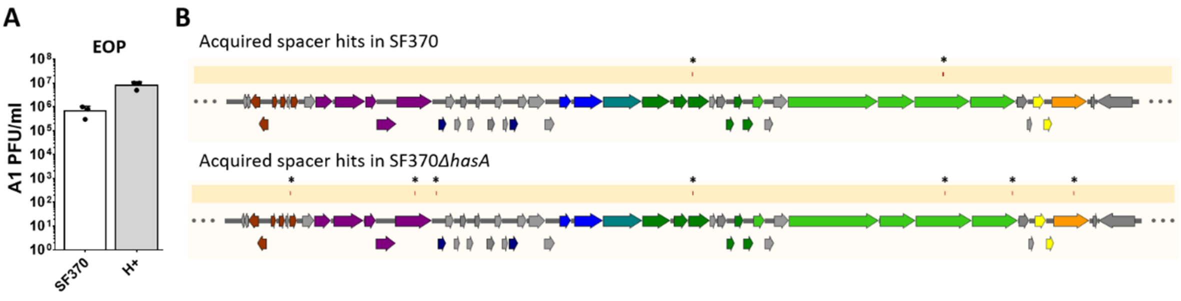

Figure 3. **(A)** Phage A1 plaquing on SF370 in the absence and presence of hyaluronidase in growth medium. Mean values ± SD from three independent experiments are shown. **(B)** Alignment of acquired spacer sequences in WT SF370 (top) or SF370*ΔhasA* (bottom) phage survivors to the Phage A1 genome highlighted in red on the dark yellow bar, marked by asterisks.
